# Dietary acid load is associated with primary osteoporosis in postmenopausal women aged 50–65 years: A cross‐sectional study

**DOI:** 10.1002/fsn3.3102

**Published:** 2022-10-13

**Authors:** Azizeh Farshbaf‐Khalili, Alireza Ostadrahimi, Javad Ahmadian Heris, Somayyeh Sarrafi, Neda Mohammadisima

**Affiliations:** ^1^ Physical Medicine and Rehabilitation Research Centre Aging Research Institute Tabriz University of Medical Sciences Tabriz Iran; ^2^ Nutrition Research Center Tabriz University of Medical Science Tabriz Iran; ^3^ Department of Allergy and Clinical Immunology Pediatric Hospital Tabriz University of Medical Sciences Tabriz Iran; ^4^ Midwifery Department Bonab Branch Islamic Azad University Bonab Iran; ^5^ Department of Biochemistry and Dietetics Faculty of Nutrition and Food Sciences Tabriz University of Medical Sciences Tabriz Iran

**Keywords:** dietary acid load, NEAP, osteoporosis, postmenopausal women, PRAL

## Abstract

This study aimed to investigate the association between dietary acid load (DAL) and primary osteoporosis. This was a cross‐sectional study. Among 850 randomly selected postmenopausal women aged 50–65 years, 232 women consisted of 124 women with normal bone mineral density (BMD) and 108 with primary osteoporosis were selected after examining the eligibility criteria. Demographic characteristics, anthropometric indices, and physical activity were collected through questionnaires. Osteoporosis was diagnosed using the dual‐energy X‐ray absorptiometry method. DAL was assessed by a valid and reliable semiquantitative food frequency questionnaire during the last year. Independent *t*‐test, Mann–Whitney, Chi‐square, and adjusted binary logistic regression were used for data analysis through SPSS/24. There were significant differences between the two groups in terms of age, body mass index (BMI), number of deliveries, and years after menopause (*p* < .05). The mean (standard deviation (SD)) potential renal acid load (PRAL) and net endogenous acid production (NEAP) were higher in postmenopausal women with osteoporosis than those with normal BMD (PRAL: −13.1 ± 11.1 mEq/day vs. −10.8 ± 12.7 mEq/day; NEAP: 29.5 ± 8.5 mEq/day vs. 31.2 ± 9.2 mEq/day). The mean consumption of potassium, magnesium, and calcium in the osteoporosis group was significantly lower than in the other group (*p* < .05). There were significant associations between osteoporosis with PRAL (odds ratio (OR) = 1.030; 95% confidence interval (CI): 1.001 to 1.060, *p* = .027) and NEAP scores (OR = 1.041; 95% CI: 1.003 to 1.081, *p* = .037). The odds of osteoporosis increased by 3% following one unit increase in PRAL score. Similarly, it increased by 4% with increasing NEAP score up to one unit. Therefore, dietary patterns that produce high DAL can have a detrimental effect on bone health.

## Graphical abstract

Some observational studies and meta‐analyses dispute the claim that acid loads generally have a detrimental effect on bone. Due to inconsistencies in the results of previous studies and the lack of sufficient evidence regarding dietary acid load (DAL) and osteoporosis in women in the early postmenopausal ages, critical ages for bone loss due to a sudden drop in estrogen level, this study as the first one in Iran aimed to investigate the relationship between DAL (included potential acid renal load and net endogenous acid production) and osteoporosis in these women.

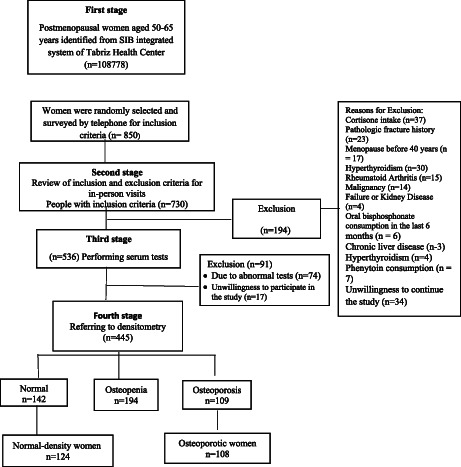

## 1 INTRODUCTION

Osteoporosis is a disease that affects millions of people around the world. In this disease, there are a decrease in bone mineral density (BMD) and destruction of the skeletal structure, and bone strength decreases (Prentice, 2004). According to the World Health Organization (WHO), osteoporosis is defined by a *T*‐score below −2.5 and normal BMD by a *T*‐score above −1.0 (Cosman et al., 2014; Prentice, 2004). Osteoporosis is a silent disease that is sometimes diagnosed with a fracture due to minor trauma or even a nontraumatic fracture (Cosman et al., 2014). It can increase morbidity and mortality. Fractures due to osteoporosis have a significant impact on the economy of the community and the family (Eastell & Hannon, 2008).

Risk factors for osteoporosis include age, genetics, gender, lifestyle (such as smoking, reduced calcium, and vitamin D intake), weight loss, and premature menopause (von Wowern, 2001). Estrogen stimulates bone production. In recent years, attention to estrogen deficiency as the pathogenesis of postmenopausal osteoporosis has increased (Haffner et al., 2021).

Several studies have shown an imbalance between the acid–base system and changes in bone density and structure (Garcia et al., 2015; Mangano et al., 2014). Mild systemic metabolic acidosis can release calcium from the bone matrix by increasing bone resorption to maintain homeostasis, thus weakening the bone and thus predisposing it to fractures (Remer et al., 2003). Dietary acid load (DAL) can affect the acid–base balance in the body. The DAL concept assumes that foods from animal sources such as cheese, fish, and meat have more acidic precursors, while fruits and vegetables are more alkaline. Low dietary pH can also affect bone health, decrease BMD, and increase bone fragility. Cross‐sectional studies show that bone can help counteract the DAL by having a detrimental effect on BMD. Both potential renal acid load (PRAL) and net endogenous acid production (NEAP) are commonly used as theoretical models to estimate the total DAL (Alexy et al., 2005; Remer et al., 2003).

The authors reported that the risk of fracture is inversely related to women's urinary citrate but positively correlated with PRAL in women (Esche et al., 2016). According to this hypothesis, a high‐acidity diet can increase the risk of osteoporosis and fractures related to decreased bone density, while consumption of alkaline‐producing foods can prevent acid‐dependent bone loss (García‐Gavilán et al., 2021). Some observational studies and meta‐analyses dispute the claim that acid loads generally have a detrimental effect on bone (De Jonge et al., 2017; Fenton et al., 2011; McLean et al., 2011). Due to inconsistencies in the results of previous studies and the lack of sufficient evidence regarding DAL and osteoporosis in women in the early postmenopausal ages, critical ages for bone loss due to a sudden drop in estrogen level, this study as the first one in Iran aimed to investigate the relationship between DAL (included PRAL and NEAP) and osteoporosis in these women.

## 2 MATERIALS AND METHODS

### 2.1 Study design and participants

**FIGURE 1:**
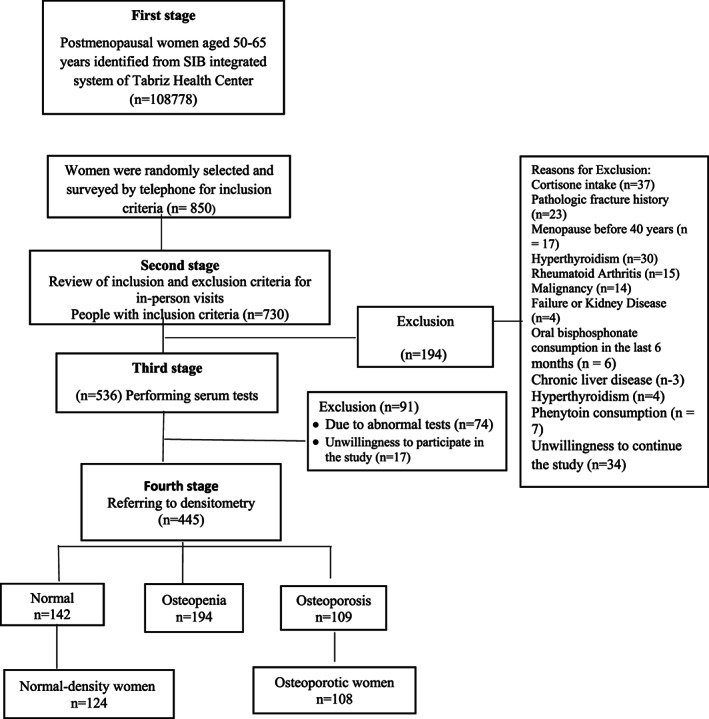
The process of selecting study participants

This is a cross‐sectional case–control study, resulting from Phase I of a mega project approved by the Tabriz University of Medical Sciences (Abdolalipour et al., n.d.; Hemmati et al., 2021). The study population was postmenopausal women aged 50–65 years who referred to Tabriz health care centers. Initially, 108,739 postmenopausal women aged 50–65 years were identified from 87 health centers through an integrated health system. Then, 850 women were randomly selected, and after examination in terms of inclusion and exclusion criteria by face‐to‐face interview, 194 women were excluded from the study due to lack of eligible criteria. Afterward, 536 women were sent to the serum biochemical testing for detecting other secondary causes of osteoporosis. Out of 536 cases, 17 were excluded due to unwillingness to participate in the study, and 74 women were excluded due to abnormal test results. Of the 445 women, 109 had osteoporosis, 194 had osteopenia, and 142 had normal BMD. Finally, 108 women with primary osteoporosis were selected as osteoporotic and 124 women with normal BMD were selected as the control group and compared in terms of social–demographic, midwifery, anthropometric, and DAL characteristics (Figure 1).

Inclusion criteria included menopause for at least 12 consecutive months, habitation in Tabriz, ability to communicate verbally to answer questions, and no history of pathological fractures during the past 10 years. Exclusion criteria included body mass index (BMI) less than 18.5, postmenopausal women under 40 years, vitamin D deficiency (<20 ng/ml), hypocalcemia, inherited diseases (hemophilia, thalassemia, hemochromatosis), use of hormonal drugs or corticosteroids during the study, drugs affecting bone metabolism including intravenous use of bisphosphonates in the last 5 years, use of oral bisphosphonates in the last 6 months, excessive use of oral bisphosphonates for more than 3 years or more than 1 month between 6 and 12 months before the study, use of parathyroid hormone analogs during the previous 12 months, renal failure, gastrointestinal diseases (such as complete gastric lavage, Crohn's disease, primary biliary cirrhosis, gastric surgery, celiac disease), bone disease other than osteoporosis, metastatic bone disease, and malignancy (Schornack & Gillies, 2003).

This study has been approved by the Ethics Committee of the Vice Chancellor for Research and Technology of Tabriz University of Medical Sciences (IR.TBZMED.REC.1397.1029). An informed consent form was signed by the participants at the beginning after explaining the method and details of the study.

### 2.2 Evaluation of study variables

The general information questionnaire was obtained by interviewing and filling out information including age, marital status, family income, vitamin D and calcium supplementation, smoking, and alcohol consumption. The midwifery questionnaire was filled with questions about the number of deliveries, the years spent after menopause, breastfeeding history, and history of injectable contraceptives. Weight and height were measured without wearing shoes with light clothing. Weight was measured using digital scales (Seca, Germany) with an accuracy of 0.1 kg. Height was measured using a stadiometer (Seca portable stadiometer) with a measurement accuracy of 0.1 cm. BMI was calculated by dividing weight (kg) by square of height (m). The international valid physical activity questionnaire‐Short Form (IPAQ‐S), which has been validated and approved in Iran, was used to measure physical activity (BashiriMoosavi et al., 2015) and the rate of metabolic equivalent of task (MET)‐min/week was calculated.

### 2.3 Bone mineral density

Anterior–posterior BMD (g/cm^2^) of the femoral neck and lumbar spine (L1–L4) was examined using Hologic QDR 4500 W dual‐energy X‐ray absorptiometry device (S/N 50266) in the densitometry center of Sina Hospital in Tabriz by an experienced and trained specialist.

### 2.4 Evaluation of dietary intake

Dietary intake was assessed by a valid and reliable 168‐item semiquantitative food frequency questionnaire (FFQ) during the past year (Remer et al., 2003). Individuals were asked to report the frequency of each food item consumption in the period in a question according to the standard serving size of that item per day, week, month, or year. To determine food groups, the items in FFQ were divided into food groups based on the type of micronutrients that make them up, including bread and cereals, fruits and vegetables, dairy products, meats and substitutes, fats, and sweets. The total values of each food were converted to grams using the guide of home scales and calculated per day for each person. The amount of energy in the FFQ items was also determined using data from the US Department of Agriculture Nutrition Table in the Nutritionist 4 software (First Databank, Inc., Hearst Corp., San Bruno, CA, USA). In cases where food items were not available in this software (such as Lavash bread, Barbari, Taftoon, and Sangak), the table of Iranian food ingredients was used.

### 2.5 DAL measurement

Then, the amount of protein, potassium, phosphorus, magnesium, and calcium was extracted to calculate PRAL and NEAP in SPSS software version 21 (SPSS Inc., Chicago IL, USA) for each person (Azar & Sarkisian, 1980; Mirmiran et al., 2010; Price et al., 1995). NEAP and PRAL scores were calculated using the following formula (Frassetto et al., 1998; Remer et al., 2003).

$$\text{NEAP}\;\left(\mathrm{mEq}/\mathrm{day}\right)=54.5\;\left[\text{protein}\;\left(g/\mathrm{day}\right)/K\;\left(\mathrm{mEq}/\mathrm{day}\right)\right]-10.2$$

$$\begin{array}{c} \text{PRAL}\;\left(\mathrm{mEq}/\mathrm{day}\right)=0.49\times \text{protein}\;\left(g/\mathrm{day}\right)+0.037\times P\;\left(\mathrm{mg}/\mathrm{day}\right)\\ \;-0.021\times K\;\left(\mathrm{mg}/\mathrm{day}\right)-0.026\times \mathrm{Mg}\;\left(\mathrm{mg}/\mathrm{day}\right)\\ \;-0.013\times \mathrm{Ca}\;\left(\mathrm{mg}/\mathrm{day}\right) \end{array}$$

### 2.6 Data analysis

In this study, to compare the qualitative variables between the case and control groups, the Chi‐Square test was used. Kolmogorov–Smirnov test was applied to determine the normality of quantitative variables. Then, an independent *t*‐test was used if it was normal and Mann–Whitney was used if it was abnormal. Also, a binary logistic regression model adjusted for confounders was used to calculate the odds ratio (OR) of osteoporosis based on PRAL and NEAP scores as continuous and dichotomous (according to the median of normal‐BMD group) variables. The Hosmer–Lemeshow test was used to test the fitness of the model. Linear regression model adjusted for confounders was applied for investigating the association of BMD with PRAL and NEAP scores. For statistical analysis of data in this study, SPSS software version 21 was used and *p*‐value less than .05 was reported as significant.

## 3 RESULTS AND DISCUSSION

The mean ± SD age and BMI of postmenopausal women in this study were 56.7 ± 4.1 year and 30.1 ± 4.8 kg/m^2^, respectively. There were significant differences between osteoporotic and normal‐BMD groups in terms of age, BMI, parity, and postmenopausal years (*p* < .05). The majority of women in the normal‐BMD group were obese but in the osteoporotic group were overweight. The mean age and parity of women in the osteoporotic were higher than the normal‐BMD group. The mean ± SD serum levels of creatinine in the osteoporotic and normal‐BMD groups were 0.93 ± 0.14 and 0.95 ± 0.15 mg/dl, respectively (*p* = .458; Table 1).

**TABLE 1:** Characteristics of postmenopausal women by BMD

Variable	Normal BMD (*n* = 124)	Osteoporosis (*n* = 108)	*p*‐Value
Age (years), mean ± SD	55.3 ± 3.8	58.2 ± 3.8	<.001
Body mass index, *N* (%)			
Normal, 18.5–24.9 kg/m^2^	8 (6.5)	14 (13.0)	<.001
Overweight, 25–29.9 kg/m^2^	35 (28.2)	59 (54.6)	
Obese, ≥30 kg/m^2^	81 (65.3)	35 (32.4)	
Postmenopausal years (years), median (IQR)	6.1 (3.7)	10.0 (4.8)	<.001
Family income, *N* (%)			
Inadequate	12 (9.7)	18 (16.8)	.234
Rather adequate	83 (66.9)	69 (64.5)	
Completely adequate	29 (23.4)	20 (18.7)	
Smoking currently or history of smoking, *N* (%)	3 (2.4)	5 (4.7)	.508
Take of supplements, *N* (%)			
Vitamin D	27 (21.8)	15 (13.9)	.295
Calcium	13 (10.5)	12 (11.1)	
Vitamin D & Calcium	24 (19.4)	17 (15.7)	
Other supplement	60 (48.4)	64 (59.3)	
Total MET (MET‐min/week) median (IQR)	401.3 (684.0)	359.3 (829.9)	.652
Marital status, *N* (%)			
Married	108 (87.1)	82 (75.9)	.065
Single, divorced, widow	16 (12.9)	25 (23.1)	
History of injectable contraceptive, *N* (%)	5 (4.3)	7 (6.7)	.312
Parity, mean ± SD	2.9 ± 1.4	3.9 ± 1.9	<.001
Lactation history, *N* (%)	112 (93.3)	100 (95.2)	.581
Serum creatinine mg/dl, mean ± SD	0.95 ± 0.15	0.93 ± 0.14	.458
BMD‐LS (g/cm^2^), mean ± SD	1.06 ± 0.21	0.71 ± 0.07	<.001
*T*‐score/ LS, median (Q25,Q75)	−0.30 (−0.7,0.3)	−2.9 (−3.5,−2.7)	<.001
*Z*‐score/ LS, median (Q25,Q75)	0.90 (0.4,1.6)	−1.6 (−2.1,−1.3)	<.001
BMD‐FN (g/cm^2^), mean ± SD	0.97 ± 0.11	0.75 ± 0.09	<.001
*T*‐score/ FN, median (Q25,Q75)	0.1 (−0.30,0.63)	−1.65(−2.2,−0.98)	<.001
*Z*‐score/ FN, median (pQ5,Q75)	0.90 (0.50,−1.9)	−0.80 (−1.3,0.0)	<.001

*Note*: Chi‐square test for categorical variables, independent *t*‐test, and Mann–Whitney for continuous.

Abbreviations: BMD, bone mineral density; IQR, interquartile range; MET, metabolic equivalent of task; SD, standard deviation.

The mean ± SD scores of PRAL and NEAP in the osteoporotic women were higher than the normal‐BMD women (PRAL: −13.1 ± 11.1 mEq/day vs. −10.8 ± 12.7 mEq/day; NEAP: 29.5 ± 8.5 mEq/day vs. 31.2 ± 9.2 mEq/day). The mean dietary intake of potassium (*p* = .010), magnesium (*p* = .006), and calcium (*p* = .030), and total fiber (*p* = .001) in the osteoporotic women were significantly lower than the other group. The difference in the intake of total energy, protein, carbohydrate, fat, and phosphorus was not statistically significant between groups (*p* > .05; Table 2).

**TABLE 2:** The score of PRAL NEAP, and some macro‐ and micronutrients among postmenopausal women by BMD

Variable	Normal BMD (*n* = 124)		Osteoporosis (*n* = 108)		*p*‐Value
	Mean ± SD	Median (IQR)	Mean ± SD	Median (IQR)	
PRAL, mEq/day	−13.1 ± 11.1	−13.2 (12.8)	−10.8 ± 12.7	−8.2 (14.1)	.020*
NEAP, mEq/day	29.5 ± 8.5	28.5 (10.3)	31.2 ± 9.2	30.9 (11.3)	.156^±^
Energy, Kcal/day	1747.6 ± 365.9	1705.2 (458.4)	1677.0 ± 377.0	1602.2 (510.4)	.150*
Protein, g/day	53.4 ± 14.2	50.6 (16.8)	50.3 ± 14.5	47.2 (17.9)	.102*
Carbohydrate, g/day	248.9 ± 55.5	240.3 (76.3)	233.9 ± 63.8	225.3 (79.0)	.056*
Fat, g/day	66.7 ± 19.8	64.9 (28.1)	64.8 ± 19.9	60.6 (26.2)	.485*
Total fiber g/day	32.3 ± 8.5	32.5 (12.2)	28.6 ± 8.8	27.3 (11.0)	.001*
Phosphorus, mg/day	1020.1 ± 280.3	967.5 (278.3)	947.2 ± 291.1	903.2 (308.2)	.053*
Potassium, mg/day	2897.1 ± 622.4	2875.0 (699.7)	2657.7 ± 788.3	2588.2 (820.1)	.010*
Magnesium, mg/day	302.1 ± 83.6	292.9 (84.0)	271.3 ± 84.4	257.9 (110.1)	.006*
Calcium, mg/day	730.8 ± 215.1	693.5 (230.9)	670.3 ± 204.8	641.9 (256.0)	.030*

Abbreviations: BMD, bone mineral density; NEAP, net endogenous acid production; PRAL, potential renal acid load.

*Independent *t*‐test, ± Mann–Whitney *U* test.

NEAP (mEq/day) = 54.5 × [protein intake (g/day)/potassium intake (mEq/day)] − 10.2.

PRAL = ([0.488 × protein in g/day] + [0.0366 × phosphorus in mg/day]) − ([0.0205 × potassium in mg/day] − [0.0263× magnesium in mg/day] − [0.0125 × calcium in mg/day]).

The results of logistic regression adjusted for age and BMI indicated that there were significant associations between osteoporosis with the PRAL as continuous (OR = 1.033; 95% CI: 1.005 to 1.050; *p* = .016) and dichotomous variable (OR = 0.375; 95% CI: 0.195 to 0.721; *p* = .003). This model adjusted for age, BMI, parity, and postmenopausal years illustrated significant associations between osteoporosis with the PRAL scores (OR = 1.030; 95% CI: 1.001 to 1.060, *p* = .027) and PRAL categories (OR = 0.358; 95% CI: 0.180 to 0.713, *p* = .003; Table 3).

**TABLE 3:** The association between PRAL and osteoporosis based on logistic regression

Variable	OR	95% Confidence interval		*p*‐Value	*B*
		Lower	Upper		
PRAL (continuous)					
Model 1^a^	1.033	1.006	1.060	.016	0.032
Model 2^c^	1.030	1.001	1.060	.027	0.030
PRAL (dichotomous)					
Model 1^b^	0.375	0.195	0.721	.003	−0.980
Model 2^d^	0.358	0.180	0.713	.003	−1.028

*Note*: Dichotomous PRAL was categorized based on median of normal group.

Abbreviations: OR, odds ratio; PRAL: potential renal acid load.

^a^ Adjusted for age and BMI, ^c^Hosmer and Lemeshow *p* = .557, Chi‐square = 8.312, *df* = 6.8; ^e^Hosmer and Lemeshow *p* = .656, Chi‐square = 5.92, *df* = 8.

^b^ Adjusted for age, BMI, postmenopausal years, parity. Hosmer and Lemeshow *p* = .375, Chi‐square = 8.625 *df* = 8;^d^ Hosmer and Lemeshow *p* = .218, Chi‐square = 10.715, *df* = 8.

Also, significant associations after adjusting for age and BMI were observed between osteoporosis with NEAP scores (OR = 1.040; 95% CI: 1.003 to 1.077, *p* = .032) and NEAP categories (OR = 0.301; 95% CI: 0.152 to 0.601, *p* = .001). These associations between osteoporosis with NEAP as continuous variable (OR = 1.041; 95% CI: 1.003 to 1.081, *p* = .037) as well as dichotomous variable (OR = 0.281; 95% CI: 0.136 to 0.578, *p* = .001) remained significant after adjusting for age, BMI, parity, and postmenopausal years (Table 4).

**TABLE 4:** The association between NEAP and osteoporosis based on logistic regression

Variable	OR	95% Confidence interval		*p*‐Value	*B*
		Lower	Upper		
NEAP (continuous)					
Model 1^a^	1.040	1.003	1.077	.032	0.039
Model 2^c^	1.041	1.003	1.081	.037	0.040
NEAP (dichotomous)					
Model 1^b^	0.301	0.152	0.601	.001	−1.197
Model 2^d^	0.281	0.136	0.578	.001	−1.271

*Note*: Dichotomous NEAP was categorized based on median of normal group.

Abbreviations: NEAP, net endogenous acid production; OR, odds ratio.

^a^ Adjusted for age and body mass index (BMI), ^c^Hosmer and Lemeshow *p* = .521, Chi‐square = 7.143, *df* = 8; ^e^Hosmer and Lemeshow *p* = .668, Chi‐square = 5.887, *df* = 8.

^b^ Adjusted for age, BMI, postmenopausal years, parity. ^d^Hosmer and Lemeshow *p* = .749, Chi‐square = 5.078, *df* = 8; ^f^Hosmer and Lemeshow *p* = .122, Chi‐square = 12.729, *df* = 8.

In addition, by using BMD as continuous variable, there was significant association between lumbar spine BMD and PRAL [adjusted β (95% CI): −0.002 (−0.004 to 0.000); *p* = .030] and also between lumbar spine BMD and NEAP scores [adjusted β (95% CI): −0.003 (−0.005 to 0.000); *p* = .030].

To the best of our knowledge, this was the first study to compare the PRAL and NEAP scores between postmenopausal women with osteoporosis and normal BMD. According to the results, the mean score of PRAL and NEAP in the osteoporotic women was higher than normal‐BMD women. The high scores of PRAL and NEAP were associated with higher odds of osteoporosis adjusted for confounders. So that, the odds of osteoporosis increased by 3% with one unit increase in PRAL score. Similarly, it increased by 4% with increasing NEAP up to one unit. The risk of osteoporosis was 65% and 72% higher in the women with the PRAL and NEAP scores above the median compared to the group with the scores below the median, respectively. Moreover, PRAL and NEAP scores correlated inversely with lumbar spine BMD.

High DAL refers to a diet that is rich in nutrients that are metabolized to noncarbonic acids such as sulfuric acid resulting from the metabolism of protein in quantities more than the amounts of alkali bicarbonate resulting from burning of organic salts e.g. potassium chloride in vegetables (Frassetto et al., 1998). Thus, long‐term consumption of such a diet might disturb the balance between HCO and CO_2_ in blood and result in chronic systemic acidosis (Hietavala et al., 2015).

It is hypothesized that chronic exposure to high DAL may be effective in reducing bone mass. Whenever systemic acidosis occurs, bone probably acts as the primary buffering system. The most important alkaline component in the body is calcium. Calcium in the blood is used to neutralize acids. Consumption of foods such as meat, cheese, fish, grains, salty foods, and legumes causes the production of acid (hydrogen ions) in the blood, which in turn leads to the release of alkaline salts from the body. Bone breakdowns and eventually the progression of osteoporosis occur (BashiriMoosavi et al., 2015; Larijani & Azadbakht, 2017; McLean et al., 2011; Remer et al., 2003; World Cancer Research Fund, 2007). Whereas sources of potassium such as vegetables contribute to a low DAL (De Jonge et al., 2017).

De Jonge et al. (2017) in a cross‐sectional study of 4672 women aged 45 and over showed that no significant associations were observed between DAL with BMD. This study is inconsistent with our study. This can be due to differences in the research population and the type of study.

In another cross‐sectional study of 1218 men and 907 women aged 60 years and older, Mangano et al. observed that there was no association between BMD and NEAP among women, PRAL is positively associated with proximal BMD of the femur (*p* trend = .04). In our study, a positive and significant relationship was observed between osteoporosis and NEAP score after adjustment for confounders in both models. As well as the results of logistic regression adjusted for age and BMI, parity, and postmenopausal years indicated that there were significant associations between osteoporosis with PRAL (in both models). This study is inconsistent with our study. In this study, men and women over 60 years of age were studied. They found that changes in calcium intake could affect the relationship between DAL and BMD (Mangano et al., 2014).

In a meta‐analysis and systematic review, the authors systematically searched for and selected studies with randomized clinical trial designs, prospective cohort studies, and meta‐analyses. The results showed that the cause‐and‐effect relationship between DAL and osteoporosis was not supported by evidence, there is no evidence that an alkaline diet can protect bone health (Fenton et al., 2011). In the mentioned article, 15 studies were included in the meta‐analysis that among them only in two studies the study outcome was BMD. None of the studies investigated the association between NEAP and PRAL with osteoporosis in a case–control methodology. Meanwhile, the age and gender of study populations were different.

In a prospective, multinational, population‐based study of 2850 children, DAL in early life was not consistently associated with bone health in childhood (Garcia et al., 2015). The reason for the contradiction of this study with our study can be the difference in the age of the research community.

According to the cross‐sectional study by McLean et al., it consisted of 5209 men and women between the ages of 28 and 62, with a total calcium intake of 800 mg per day. In this study, in older men, the acid load diet was inversely related to the BMD of the femoral neck based on NEAP estimates. But in this group of older men, there was no relationship between NEAP and PRAL with lumbar spine BMD. Estimated NEAP and PRAL were not associated with BMD at any site among offspring cohort men and women and original cohort women (McLean et al., 2011). The reason for the difference in the results of this study with our study could be that in this study, women were included in the study from all age groups and that one of the criteria for inclusion in the study was calcium intake of <800 mg/day. The method of study was also different from our study.

The mean dietary intake of potassium, magnesium, and calcium in the osteoporotic women was significantly lower than the other group. The difference in the intake of protein and phosphorus was not statistically significant between groups in the present study. In the study of García‐Gavilán et al. (2021), calcium, protein, magnesium, and phosphorus intake was higher in the osteoporosis group, but potassium intake was significantly lower (*p* < .05). The reason for the possible difference in the inclusion criteria could be that the participants in the study were adults 55–80 years old at high risk of cardiovascular disease or overweight or obese people with metabolic syndrome. In two other studies, the positive effects of calcium and the negative effects of protein on bone have been shown (Bonjour, 2005; Tylavsky et al., 2008).

Trace minerals may play an important role in the maintenance of bone quality because they act as metalloenzymes in the synthesis of collagen and other proteins which constitute the bone structure. The risk of nutritional deficiencies, especially trace elements and vitamins, is high during menopause (Gür et al., 2002). Mg acts as a substitute for calcium in transportation and mineralization processes (Center & Eisman, 1997), additionally, it has direct effects on the bone formation processes and mineral aggregation. Mg also does a large number of other functions, comprising modulation of the growth factors, hormones, cytokine actions, and enzyme cofactor function (Wallach, 1991). The effects of calcium, magnesium, potassium, and other minerals on bone mineralization have been known for decades. Clinical studies show that potassium plays a central role in determining whether a person's diet produces a pure acid or an alkaline substance. More potassium intake has been positively associated with bone metabolism. Interstitial bone fluid has higher concentrations of potassium and sodium and lower concentrations of calcium and phosphorus compared to bone or plasma crystals. The interstitial fluid content of bone is directly related to the amount of potassium consumed. The first line of defense is skeletal protection in the buffering of metabolic acids. Therefore, if potassium intake is higher regardless of the food source, then adaptations are created that affect not only calcium balance but also other metabolic indicators of bone metabolism (Gür et al., 2002; Tylavsky et al., 2008).

This study has several strengths. To the best of our knowledge, we were the first to investigate the relation of DAL with postmenopausal osteoporosis comparing two osteoporotic and normal‐BMD women. Because early years after menopause is an important time to start or accelerate osteoporosis due to a sudden drop in estrogen, preventive measures at this age are important to prevent its complications, especially fractures in old age. Therefore, this study was performed on postmenopausal women aged 50–65 years. However, the results of the study cannot be generalized to the other ages and to men. Because this study is an observational one, the existence of a relationship certainly does not indicate a cause‐and‐effect relationship. Other weaknesses of the study include the age limit of 50–65 years for participants and the impossibility of examining postmenopausal women over 65 years of age. Lack of matching groups according to age and BMI was another limitation that was adjusted by statistical methods. In this study, we estimated PRAL and NEAP but did not perform renal function tests, except for serum creatinine. It is recommended that renal function tests be performed in future studies. In the future, intervention studies are needed to clarify the role of DAL in the prevention of osteoporosis in postmenopausal women.

This study was conducted on people with osteoporosis and normal BMD. It is suggested that in future studies, people with osteopenia should also be included in the study and investigated.

## 4 CONCLUSION

In conclusion, the mean score of PRAL and NEAP in the osteoporotic women was higher than normal‐BMD women. So that, the odds of osteoporosis increased by 3% with one unit increase in PRAL score. Similarly, it increased by 4% with increasing NEAP up to one unit. The risk of osteoporosis was 65% and 72% higher in the women with the PRAL and NEAP scores above the median compared to the group with the scores below the median, respectively. These findings imply that dietary patterns that produce high DAL can have a detrimental effect on bone health.

## FUNDING INFORMATION

This article was financially supported by the Tabriz University of Medical Sciences (Grant no.: 62807).

## CONFLICT OF INTEREST

The authors declare that they have no conflict of interest.

## ETHICAL STATEMENT

This study does not involve any human or animal testing.
